# Increased percentage of PD-L1^+^ natural killer cells predicts poor prognosis in sepsis patients: a prospective observational cohort study

**DOI:** 10.1186/s13054-020-03329-z

**Published:** 2020-10-19

**Authors:** Wenqiang Jiang, Xusheng Li, Miaoyun Wen, Xiaoyu Liu, Kangrong Wang, Qiaosheng Wang, Ya Li, Maohua Zhou, Mengting Liu, Bei Hu, Hongke Zeng

**Affiliations:** 1 Department of Emergency and Critical Care Medicine, Guangdong Provincial People’s Hospital, Guangdong Academy of Medical Sciences, 106 Zhongshan Er Road, Guangzhou, 510080 Guangdong China; 2 The Second School of Clinical Medicine, Southern Medical University, 1063 Shatai Nan road, Guangzhou, 510515 China; 3 Department of Critical Care Medicine, The First Affiliated Hospital, University of South China, No. 69, Chuanshan Road, Hengyang, 421001 Hunan China; 4 School of Medicine, South China University of Technology, Guangzhou, 510006 China; 5 Division of Laboratory, Guangdong Provincial People’s Hospital, Guangdong Academy of Medical Sciences, 106 Zhongshan Er Road, Guangzhou, 510080 Guangdong China

**Keywords:** Sepsis, NK cells, PD-L1, Biomarker, Mortality, Prognosis

## Abstract

**Background:**

Natural killer (NK) cells play a major role in immune tolerance after sepsis, and the programmed cell death 1 (PD-1) and programmed cell death ligand 1 (PD-L1) system mediates evasion of host immunity. The correlation between PD-L1 levels in NK cells and the prognosis of patients with sepsis, however, has not been elucidated. Thus, it was hypothesized that PD-L1 in NK cells could be a novel biomarker of the mortality for sepsis patients.

**Methods:**

A prospective, observational, cohort study in a general intensive care unit had earlier enrolled patients according to the sepsis-3 criteria, and peripheral blood samples were collected within 24 h post-recruitment. The expression of four co-signaling molecules (PD-1, CD28, PD-L1, and CD86) in NK cells was assayed, and the sequential organ failure assessment (SOFA) scores were recorded on day 1. Patients were followed up until 28 days. Multivariate regression analysis assessed the independent risk factors for 28-day mortality. The association between biomarkers and 28-day mortality was assessed by Cox regression survival analysis. The accuracy of biomarkers for mortality was determined by the area under the receiver operating characteristic (ROC) curve (AUC) analysis.

**Results:**

A total of 269 patients were recruited, and 114 patients were finally included for final analysis. Of these, 30 (26.3%) patients died during 28 days. The percentage of PD-L1^+^ NK cells (OR 1.022; 95% CI 1.002–1.043) and SOFA scores (OR 1.247; 95% CI 1.092–1.424) were independent risk factors for 28-day mortality. The AUC of the percentage of PD-L1^+^ NK cells, SOFA scores, and their combination model were 0.655 (0.559–0.742), 0.727 (0.635–0.807) and 0.808 (0.723–0.876), respectively. The combination model was the indicator with the best AUC to predict mortality in 28 days (all *p* < 0.05). Patients with the percentage of PD-L1^+^ NK cells above the cutoff point 5.58% (hazard ratio (HR) 10.128 (1.372–74.772), *p* = 0.001), and the combination model prediction possibility above 0.1241 (HR 13.730 (3.241–58.158), *p* < 0.001) were the indexes that had greater discriminative capacity to predict 28 days mortality.

**Conclusions:**

The percentage of PD-L1^+^ NK cells at admission serves as a novel prognostic biomarker for predicting mortality and contributes to improve the predictive capacity of SOFA score in patients with sepsis.

## Background

The incidence of sepsis is high and the condition remains one of the leading causes of death [1]. Indeed, it accounts for 37.3% of all intensive care unit (ICU) patients in China and is responsible for 66.7–79 deaths/100,000 individuals [2–4]. The risk stratification of sepsis patients is conducive to timely measures to improve the prognosis of patients. As a predictor of sepsis prognosis, the biomarkers of immune function may be valuable for clinical application [1].

Immunosuppression is one of the main mechanisms leading to poor prognosis of sepsis [1]. Natural killer (NK) cells, as innate lymphoid cells [5], play a key role in the process of anti-infectious immunity. NK cells may play a protective role in the process of bacterial infection by releasing cytokines such as interferon-γ (IFN-γ) and granulocyte-macrophage colony stimulating factor (GM-CSF) [6, 7].

Recent experimental studies have found that NK cells play a critical role in immune tolerance after sepsis [8]. However, there is only a modicum of information about the role of NK cells in the progression of sepsis. The activation of NK cells is controlled by a balance between signals transmitted through inhibition and activation of receptors [9]. It has been reported that a shift toward inhibitory receptors and impaired effector functions on NK cells contribute to the immunosuppression during sepsis [10]. The immunosuppressive molecules, such as PD-L1, with increased expression in the innate myoid cells, are one of the important mechanisms underlying immunosuppression in sepsis [11]. As a critical component of the innate immune system-innate lymphoid cells, it has remained uncertain whether NK cell PD-L1 is involved in human sepsis immunosuppression.

Thus, the present study aimed to evaluate the expression of PD-L1 in NK cells on the prognosis of patients with sepsis according to sepsis-3 criteria.

## Methods

### Design, setting, and patients

A prospective observational study was conducted by enrolling consecutive sepsis patients. The study was approved by the Guangdong Provincial People’s Hospital Ethics Committee in accordance with the Declaration of Helsinki in a general ICU of Guangdong Provincial People’s Hospital. Informed consent was obtained from all patients or their legal proxy before enrollment. The study was performed from February 2017 to May 2019. The inclusion criteria were sepsis patients, aged ≥ 18 years old, who met the sepsis 3.0 criteria. Patients were excluded if any of the following criteria were fulfilled: end-stage of chronic disease and estimated survival time < 28 days, with autoimmune disease, tumor, immunodeficiency, or long-term use of immune suppressants, the onset time was > 5 days, and consent could not be obtained. All patients were evaluated in the ICU on day 1 (within 24 h after admission) and administered conventional therapy according to the 2016 international guidelines for the management of severe sepsis and septic shock [12].

### Data and sample collection

Baseline characteristics, including demographic data, the site of infection, preexisting clinical conditions, organ function, and disease severity, were recorded within 24 h after satisfying the criteria for sepsis. The severity of the disease was assessed using the Acute Physiology and Chronic Health Evaluation II (APACHE II) [13] and sequential organ failure assessment (SOFA) scores [14]. The patients were followed for at least 28 days after enrollment. According to the mortality within 28 days, the patients were divided into non-survival and survival groups. EDTA anticoagulated peripheral venous blood samples were withdrawn on day 1.

### Flow cytometry

The expression of PD-1, CD28, PD-L1, and CD86 in NK cells was assayed in the whole peripheral blood samples via flow cytometry within 1 h of blood collection. The cells were stained with monoclonal antibodies and isotype controls according to the manufacturer’s recommendations (anti-human PD-1, PD-L1, and CD86; BD Pharmingen, Franklin Lake, NJ, USA; The other antibodies listed below were obtained from Biolegend, San Diego, CA, USA). The other antibodies listed below were obtained from San Diego, CA, USA. Aliquots of 100 μL of whole blood were incubated with PerCP/cyanine5.5-labeled anti-CD3 (5 μL clone HIT3a), APC/cyanine7-labeled anti-CD56 (5 μL clone HCD56), APC-labeled anti-PD-1 (20 μL clone MIH4), PE.Cy7-labeled anti-CD28 (5 μL clone CD28.2), PerCP/Cy5.5-labeled anti-CD3 (5 μL clone HIT3a), APC/Cy7-labeled anti-CD56 (5 μL clone HCD56), APC-labeled anti-PD-L1 (20 μL clone MIH1), and PE.Cy7-labeled anti-CD86 (20 μL clone 2331 (FUN-1)). The erythrocytes were lysed, and cells evaluated by a researcher blinded to our clinical data. The samples were processed on CytoFLEX (Beckman Coulter Inc. Brea, CA, USA) and analyzed using CytExpert software version 2.0 (Beckman Coulter Inc.). The lymphocytes were gated by forward scatter (FSC) and side scatter (SSC), and NK cells subsets were further identified by CD3^−^ and CD56^+^ staining.

### Enzyme-linked immunosorbent assay (ELISA)

IFN-γ and GM-CSF levels of the peripheral venous blood plasma were quantified using ELISA kits (CUSABIO TECHNOLOGY LLC, Houston, TX, USA), according to the manufacturer’s protocols.

The number of peripheral blood lymphocytes and NK cells was calculated by the following formula: Peripheral blood lymphocyte count = total white blood cell count × (lymphocytes/leukocytes). Peripheral blood NK cell count = blood lymphocyte count × (NK cells/lymphocytes).

### Statistical analysis

MedCalc (Version 15.2.2, Ostend, Belgium) was used for receiver operating characteristic (ROC) curve analysis, and SPSS (version 20.0, SPSS Inc., Chicago, IL, USA) was applied for other analyses. The baseline characteristics were described as frequencies, percentages, median, and inter-quartile ranges. The comparisons between groups were made using the Pearson’s *χ*^2^ test for categorical data and non-parametric Mann–Whitney *U* test for continuous variables. The binary logistic regression was used to identify the variables associated with 28-day mortality and hospital mortality in patients with sepsis. Next, we constructed a regression equation model for the prognosis of sepsis. After multivariate analysis, the variables were stratified using the optimal threshold indicated by the ROC curve. The patient survival was analyzed using Cox regression analysis. All statistical tests were two-tailed, and *p* < 0.05 was considered statistically significant.

## Results

### Patient characteristics

**Fig. 1:**
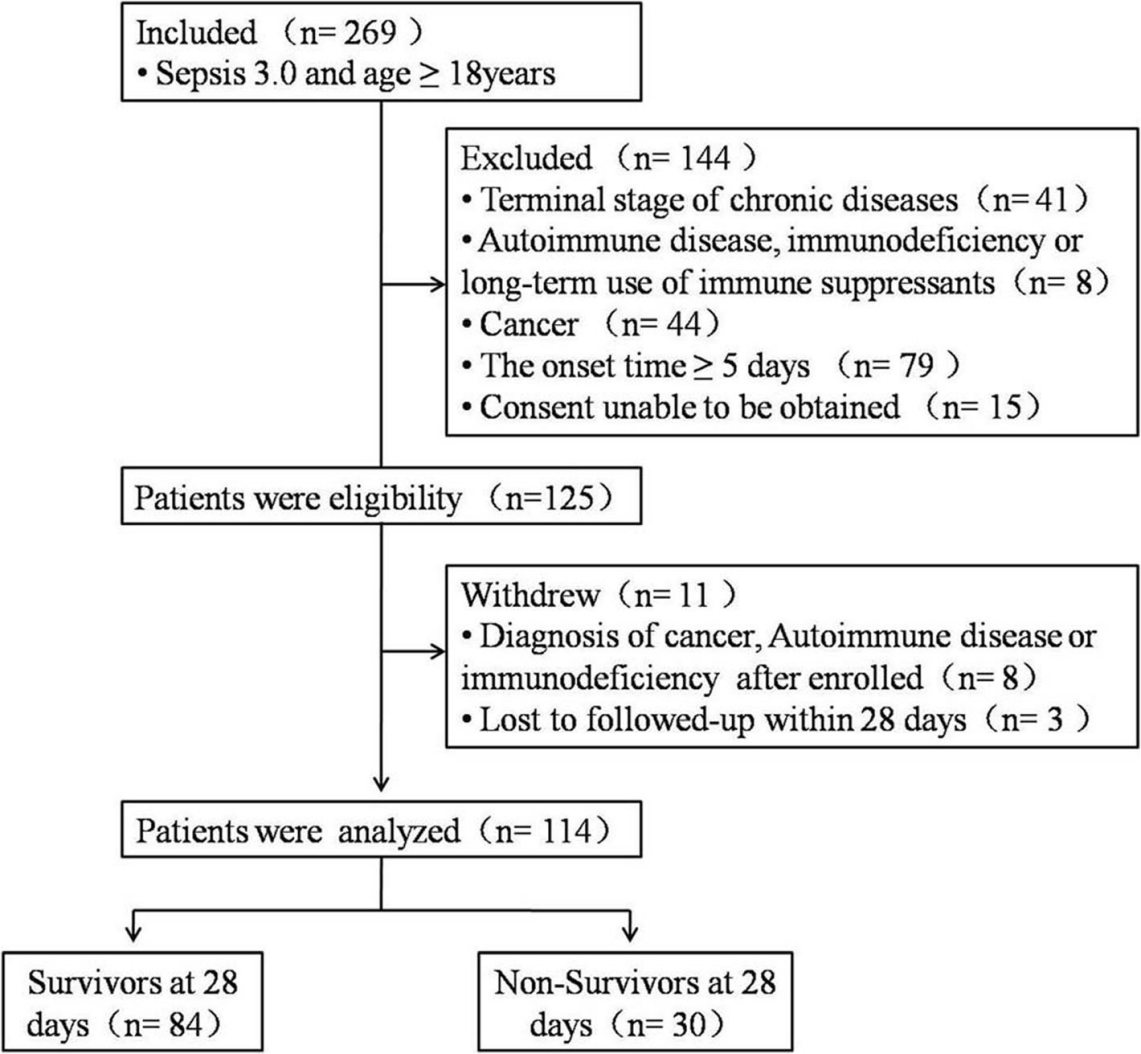
Schematic of the study

In total, 269 adult patients (all Chinese) with sepsis were admitted to the ICU of Guangdong Provincial People’s Hospital. Based on prespecified criteria, 144 patients were excluded, and 11 patients withdrew from the study. Finally, 114, including 73 males and 41 females, completed the study. Of these, 30 died within 28 days (Fig. 1). The cohort comprised 49 patients who were bacteriology-positive; 81.6% (93/114) of patients were detected by blood culture: 15.1% (14/93) had positive cultures and 84.9% (79/93) negative cultures. Among those with positive blood cultures, 21.4% (3/14) were isolated from patients with bloodstream infection, 21.4% (3/14) with pulmonary infection, 28.6% (4/14) with abdomen infection, 14.3% (2/14) with urinary system infection, and 14.3% (2/14) with other infections. Twenty-eight patients had nosocomial infections, and 41 patients had septic shock. APACHE II scores (22 [15–26] vs.15 [10–22], *p* = 0.003), SOFA scores (9 (6–13) vs. 6 (4–9), *p* < 0.001), lactate level (2.5 (1.7–4.0) mmol/L vs. 1.5 (1.1–2.5) mmol/L, *p* = 0.002), and positive rate of bloodstream infection (16.7% vs. 4.8%, *p* = 0.038) were significantly higher in the non-survivors than the survivors. However, the Glasgow Coma Scale (GCS) scores showed the opposite trend between the two groups (7 (6–14) vs.14 (8–15), *p* = 0.007). No significant differences were detected between the survivor and the non-survivor groups with respect to age, gender, bacteriology-positive, the number of white blood cells, body temperature, heart rate, and mean arterial pressure in 28-day mortality. A summary of patient demographics and measurements is presented in Table 1.

**Table 1:** Baseline characteristics of the patients according to 28-day survival

Parameters	All patients *n* = 114	Non-survivors *n* = 30	Survivors *n* = 84	*p* value
**Demographic characteristics**				
Female, *n* (%)	41 (36.0)	11 (36.7)	30 (35.7)	0.926
Age (years)	66 (55–79)	64 (53–81)	68 (56–78)	0.483
**Laboratory findings**				
WBC (× 10^9^/L)	14.66 (9.26–21.23)	15.26 (8.08–20.13)	14.40 (9.49–21.48)	0.625
Lac (mmol/L)	1.64 (1.20–2.70)	2.5 (1.7–4.0)	1.5 (1.1–2.5)	0.002
CRP (mg/L)	153.3 (84.7–200.1)	164.3 (101.6–200.1)	140.8 (75.1–200.1)	0.536
PCT (ng/ml)	25.5 (6.8–72.3)	35.0 (17.8–108.0)	23.3 (5.4–54.7)	0.077
Bacteriology positive, *n* (%)	49 (43.0)	14 (46.7)	35 (41.7)	0.635
**Vital signs**				
T (°C)	36.8 (36.5–37.2)	36.8 (36.5–37.5)	36.8 (36.5–37.2)	0.400
Heart rate (bpm)	97 (84–111)	102 (94–113)	92 (81–110)	0.042
MAP (mmHg)	87 (79–98)	84 (73–94)	88 (81–99)	0.114
**Source of infection,** ***n*** **(%)**				
Bloodstream	9 (7.9)	5 (16.7)	4 (4.8)	0.052
Lungs	40 (35.1)	14 (46.7)	26 (31.0)	0.122
Abdomen	39 (34.2)	7 (23.3)	32 (38.1)	0.143
Urinary system	18 (15.8)	2 (6.7)	16 (19.0)	0.148
other	8 (7.0)	2 (6.7)	6 (7.1)	1.000
**Severity of illness**				
GCS score	13 (6–15)	7 (6–14)	14 (8–15)	0.007
APACHE II score	17 (12–24)	22 (15–25)	15 (10–22)	0.002
SOFA score	7 (5–10)	9 (6–13)	6 (4–9)	< 0.001

Data are shown as median and interquartile range unless otherwise indicated. Pearson’s chi-square test was performed for sex and bacteriology positive, while the non-parametric test of two independent samples (Mann–Whitney) was for the others

*WBC* white blood cell, *Lac* lactate, *GCS* Glasgow coma scale, *APACHE II* Acute Physiology and Chronic Health Evaluation, *SOFA* sepsis-related organ failure assessment, *T* temperature, *MAP* mean arterial pressure

### Comparison of blood cell, lymphocyte subsets, and co-signaling molecules expression on NK cells between survivors and non-survivors

The counts of lymphocytes (0.44 (0.29–1.04) vs. 0.81 (0.48–1.13) × 10^9^/L, *p* = 0.011), T lymphocytes (0.16 (0.10–0.33) vs. 0.32 (0.14–0.70) × 10^9^/L, *p* = 0.012), and NK cells (0.03 (0.01–0.07) vs. 0.07 (0.03–0.12) × 10^9^/L, *p* = 0.006) were significantly lower in the non-survivors than the survivors.

**Fig. 2:**
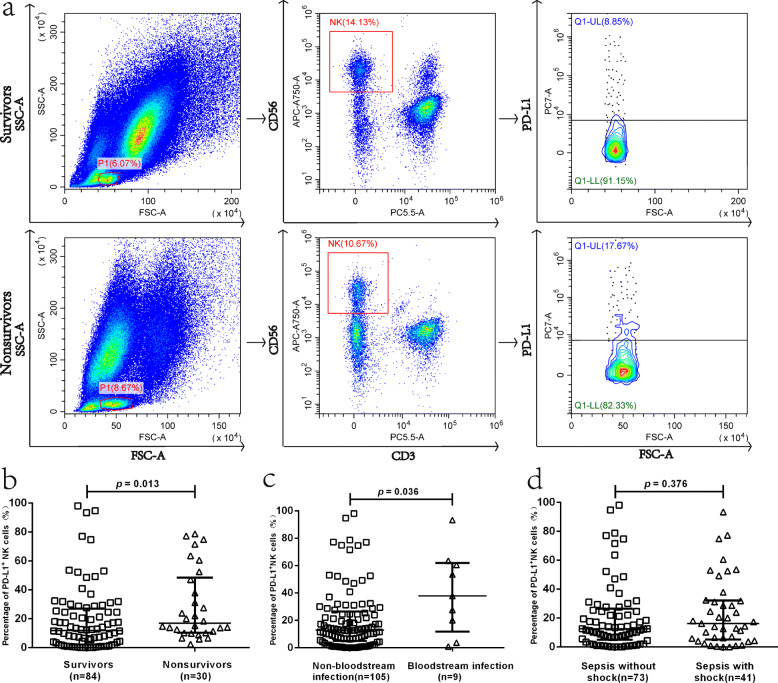
**a**–**d** Percentage of PD-L1^+^ NK cells in the derivation cohort of patients with sepsis. **a** Flow dot plots of the percentage of comparison PD-L1^+^ NK cells between non-survivor and survivors of sepsis patients. **b** Dot plots showing the percentage of PD-L1^+^ NK cells of sepsis patients to ascertain the statistical differences between the non-survivor and the survivor groups. The samples had a significantly higher proportion of PD-L1^+^ (16.77 (10.20–48.42) vs. 11.84 (2.01–26.98), *p* = 0.013) in NK cells in non-survivors (*n* = 30) as compared to survivors (*n* = 84). **c** Dot plots showing the percentage of PD-L1^+^ NK cells of sepsis patients to ascertain the statistical differences between the bloodstream infection and non-bloodstream infection group. The samples had a significantly higher proportion of PD-L1^+^ [37.77 (11.83–61.98) vs. 12.55 (4.99–26.44), *p* = 0.036] in the NK cells in the bloodstream infection (*n* = 9) as compared to the non-bloodstream infection group (*n* = 105). **d** Dot plots showing the percentage of PD-L1^+^ NK cells of sepsis patients to ascertain the statistical differences between patients with and without septic shock. No significant difference was detected in the percentage of PD-L1^+^ NK cells between the two groups

The percentage of PD-L1^+^ NK cells (16.77 (10.20–48.42) vs. 11.84 (2.01–26.98)%, *p* = 0.013) (Fig. 2a) and the mean fluorescence intensity (MFI) of CD28 in NK cells (4738 (3333–20,171) vs. 12,416 (5913–21,683), *p* = 0.036) was significantly higher in the non-survivors than the survivors. No significant differences were detected between the survivor and non-survivor groups with respect to the positive percentages of PD-1, CD28, CD86, MFI of PD-1, PD-L1, CD86, and PD-1/PD-L1 in NK cells (Table 2).

**Table 2:** Comparison of blood cell analysis, cell count of NK, and expression of co-signaling molecules in NK cells of the survival and non-survival groups according to 28-day mortality [10^9^/L, Md (IQR)]

Parameters	All patients (*n* = 114)	Non-survivors (*n* = 30)	Survivors (*n* = 84)	*p* value
L (× 10^9^/L)	0.69 (0.4–1.12)	0.44 (0.29–1.04)	0.81 (0.48–1.13)	0.011
T cells (× 10^9^/L)	0.28 (0.13–0.52)	0.16 (0.10–0.33)	0.32 (0.14–0.70)	0.012
NK cells (× 10^9^/L)	0.06 (0.02–0.11)	0.03 (0.01–0.07)	0.07 (0.03–0.12)	0.006
CD28 in NK (%)	4.38 (1.64–9.96)	4.95 (2.07–10.68)	4.30 (1.41–9.45)	0.336
PD-1 in NK (%)	2.13 (0.91–4.56)	1.94 (0.93–4.97)	2.26 (0.81–4.28)	0.862
CD86 in NK (%)	2.82 (1.16–5.36)	3.08 (1.53–6.38)	2.59 (0.97–5.34)	0.332
PD-L1 in NK (%)	13.50 (5.28–28.79)	16.77 (10.20–48.42)	11.84 (2.01–26.98)	0.013
PD-1/PD-L1 in NK	0.12(0.04–0.8)	0.13(0.04–1.01)	0.09(0.04–0.29)	0.195
MFI of CD28 in NK	11,027 (4381–21,608)	4738 (3333–20,171)	12,416 (5913–21,683)	0.036
MFI of PD-1 in NK	2652 (2064–3417)	2685 (1975–4043)	2652 (2114–3403)	0.953
MFI of CD86 in NK	2627 (2057–3368)	2666 (1845–3240)	2610 (2095–3389)	0.767
MFI of PD-L1 in NK	32,331 (13,285–66,937)	26,192 (11,165–70,708)	34,562 (14,490–66,761)	0.361

Data are shown as median and interquartile range unless otherwise indicated. All the above were performed on a non-parametric test of two independent samples (Mann–Whitney)

*L* lymphocyte counts, *T cells* T cells counts, *NK cells* NK cells counts, *PD-1* programmed cell death-1, *PD-L1* programmed cell death-Ligand 1, *MFI* mean of fluorescence intensity

### Correlation between the percentage of PD-L1^+^ NK cells and 28-day mortality, bloodstream infection, and shock

The percentage of PD-L1^+^ NK cells on ICU admission was significantly increased in the non-survivor as compared to the survivor groups (Fig. 2a, b); also, the number of septic patients with bloodstream infection showed a significant increase in percentage of PD-L1^+^ NK cells in comparison with that in patients with non-bloodstream infection (Fig. 2c). However, no significant difference was detected in the percentage of PD-L1^+^ NK cells between the sepsis with and without shock (Fig. 2d).

### Independent predictor of 28-day mortality in sepsis patients

The parameters that exhibited significant differences by univariate analysis between survivors and non-survivors, which including lactate level, counts of lymphayte, T lymphayte, and NK cells, bloodstream infection, the percentage of PD-L1^+^ NK cells, MFI of CD28 in NK, GCS score, SOFA score, and APACHE II score, were included in multivariate logistic regression analysis. The results showed that the percentage of PD-L1^+^ NK cells (odds ratio (OR) 1.022, 95% CI 1.001–1.042; *p* = 0.039) and SOFA score (OR 1.263, 95% CI 1.107–1.441; *p* = 0.001) were independently associated with 28-day mortality. The detailed data are presented in Table 3. A predicted probability of regression equation model was established as follows:

$$ y=1/\left(1+{\mathrm{e}}^{0.021\ast \mathrm{PD}-\mathrm{L}1\ \mathrm{in}\ \mathrm{NK}+0.234\ast \mathrm{SOFA}\ \mathrm{score}-3.441}\right) $$

**Table 3:** Multivariate analysis of independent risk factors for 28-day mortality

Variable	B	SE	Wald	*p* value	Odds ratio	95% CI for odds ratio	
						Lower limit	Upper limit
NK PD-L1	0.021	0.010	4.251	0.039	1.022	1.001	1.042
SOFA score	0.234	0.067	12.089	0.001	1.263	1.107	1.441
Constant	− 3.441	0.703	23.936	0.000	0.032		

*SOFA* sepsis-related organ failure assessment, *NK PD-L1* positive percentage of PD-L1 in NK cells

### Predictive performance of co-signaling molecules in NK, clinical severity, the combined model, and inflammatory biomarkers for 28-day mortality in sepsis patients

**Fig. 3:**
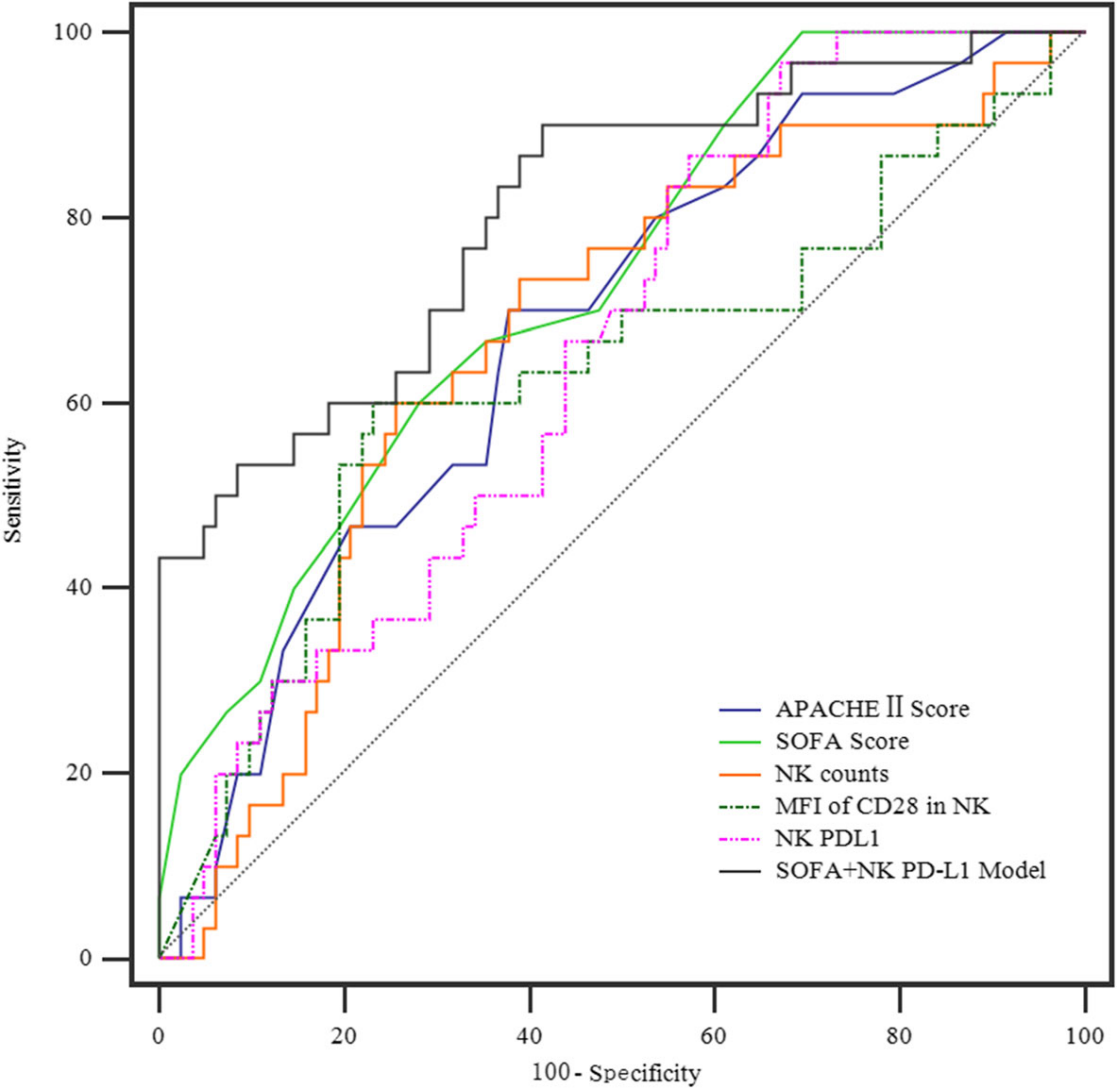
ROC analyses for predicting 28-day mortality. Comparison of AUROC: SOFA+NK PD-L1 model vs. APACHE II score (*p* = 0.048); SOFA+NK PD-L1 model vs. SOFA score (*p* = 0.037); SOFA+NK PD-L1 model vs. NK counts (*p* = 0.038); SOFA+NK PD-L1 model vs. MFI of CD28 in NK (*p* = 0.043); SOFA+NK PD-L1 model vs. NK PD-L1 (*p* = 0.046). APACHE II, Acute Physiology and Chronic Health Evaluation II; SOFA, sepsis-related organ failure assessment; NK PD-L1, the percentage of PD-L1^+^ NK cells; AUROC, area under the ROC curve; ROC, receiver operating curve

The area under the ROC curve (AUC) of the positive percentage of PD-L1, MFI of CD28 in NK cells, and the number of NK cells for predicting 28-day mortality were 0.655 (0.559–0.742), 0.630 (0.534–0.719), and 0.670 (0.574–0.755), respectively. Moreover, the SOFA score was 0.727 (0.635–0.807) and the APACHE II score was 0.678 (0.583–0.763) (Fig. 3). The AUC of the combination of SOFA score and positive percentage of PD-L1 in NK cells was 0.808 (0.723–0.876). The comparison of the AUC of SOFA+NK PD-L1(the percentage of PD-L1^+^ NK cells) model vs. APACHE II score (0.808 vs. 0.678, *p* = 0.048), SOFA+NK PD-L1 model vs. SOFA score (0.808 vs. 0.727, *p* = 0.037), SOFA+NK PD-L1 model vs. NK counts (0.808 vs. 0.0.670, *p* = 0.038), SOFA+NK PD-L1 model vs. MFI of CD28 in NK cells (0.808 vs. 0.630, *p* = 0.043), and SOFA+NK PD-L1 model vs. NK PD-L1 (0.808 vs. 0.655, *p* = 0.046) indicates that the mortality prediction of the SOFA+NK PD-L1 model was better than the other isolated indicator. It did not reveal any statistically significant difference among the other indicators on day 1. The 28-day mortality was predicted according to the cutoff, and the sensitivity and the specificity were shown in Table 4. The AUC of C-reactive protein (CRP) d3, procalcitonin (PCT) d3, and PCT d5 for predicting 28-day mortality was 0.684 (0.526–0.843), 0.720 (0.568–0.872), and 0.724 (0.560–0.889), respectively. PCT and PD-L1 in NK cells were detected along with SOFA score which was assessed on d1 in 87 patients simultaneously; among them, 23 patients were from the 28-day non-survival group and 64 patients from the survival group. The AUC of SOFA + NK PD-L1 model was significantly larger than that of PCT + NK PD-L1 model [0.836 (0.742–0.907) vs. 0.679 (0.571–0.775), *p* = 0.032].

**Table 4:** The percentage of PD-L1+ NK cells, SOFA score, the combination of them and NK counts, MFI of CD28 in NK, and APACHE II score for predicting 28-day mortality

Variables	ROC curve			Sensitivity (%)	Specificity (%)	Youden index (%)	PPV (%)	NPV (%)
	AUC (95% CI)	Best cutoff	*p*					
APACHE II score	0.678 (0.583–0.763)	18	0.001	70.00	62.20	32.20	40.39	85.00
SOFA score	0.727 (0.635–0.807)	8	< 0.001	60.00	71.95	31.95	43.91	83.10
NK counts (× 10^9^/L)	0.670 (0.574–0.755)	≤ 0.029	0.003	60.00	74.39	34.39	41.16	83.56
MFI of CD28 in NK	0.630 (0.534–0.719)	≤ 5390	0.046	60.00	76.38	36.83	48.65	84.00
NK PD-L1 (%)	0.655 (0.559–0.742)	5.58	0.004	96.67	32.93	29.59	34.53	96.43
SOFA+NK PD-L1 model	0.808 (0.723–0.876)	0.1241	< 0.001	90.00	58.54	48.54	44.27	94.12

*APACHE II* Acute Physiology and Chronic Health Evaluation II, *SOFA* sepsis-related organ failure assessment, *NK PD-L1* the percentage of PD-L1^+^ NK cells

### Survival

**Fig. 4:**
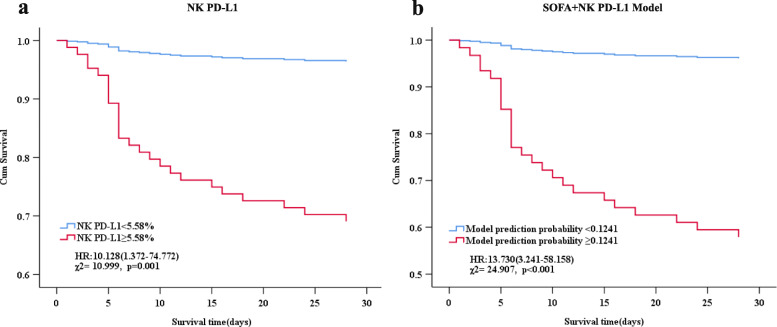
Survival curves. **a** Cox regression analysis survival curves showed that septic patients with the percentage of PD-L1^+^ NK cells  ≥ 5.58% had higher mortality at 28 days (HR 10.128 (1.372–74.772), *χ*^2^ = 10.999; *p* = 0.001) as compared to patients with lower levels. **b** Septic patients with the predicted probability of the SOFA+NK PD-L1 model  ≥ 0.1241 had higher mortality at 28 days (HR 13.730 (3.241–58.158), *χ*^2^ = 24.907; *p* < 0.001) as compared to those with lower levels. SOFA, sepsis-related organ failure assessment; NK PD-L1, the percentage of PD-L1^+^ NK cells; HR, hazard ratio

Cox regression analysis survival analysis showed a higher percentage of PD-L1^+^ NK cells in septic patients than the cutoff of 5.58% and, hence, higher mortality at 28 days (hazard ratio (HR) 10.128 (1.372–74.772), *χ*^2^ = 10.999; *p =* 0.001) as compared to patients with lower percentage of PD-L1^+^ NK cells (Fig. 4a). Additionally, we used the regression equation model to estimate the SOFA score and the percentage of PD-L1^+^ NK cells to calculate the predicted probability. The results showed that the predicted probability of model of SOFA score and the percentage of PD-L1^+^ NK cells were higher than the cutoff of 0.1241 with higher mortality at 28 days (HR 13.730 (3.241–58.158), *χ*^2^ = 24.907; *p <* 0.001) as compared to patients with lower predicted probability (Fig. 4b).

### Correlation analysis of plasma IFN-γ and GM-CSF levels, clinical severity, and count of NK cells, and percentage of PD-L1^+^ NK cells

No significant associations were detected between the count of NK cells/the percentage of PD-L1^+^ NK cells and either plasma IFN-γ and GM-CSF levels, APACHE II, or SOFA scores on day 1 (*p* > 0.05). However, the percentage of PD-L1^+^ NK cells was positively correlated with APACHE II score d2 (Spearman’s *ρ* = 0.251, *p* = 0.037), APACHE II score d3 (Spearman’s *ρ* = 0.280, *p* = 0.037), APACHE II score d6 (Spearman’s *ρ* = 0.296, *p* = 0.037), SOFA score d2 (Spearman’s *ρ* = 0.289, *p* = 0.017), SOFA score d3 (Spearman’s *ρ* = 0.289, *p* = 0.039), and SOFA score d6 (Spearman’s *ρ* = 0.350, *p* = 0.015).No significant differences were detected between groups with higher NK cell count and lower NK cell count with respect to the plasma IFN-γ [10.96 (4.71–27.78) vs 13.18 (2.25–26.99), *p* = 0.915] and GM-CSF [0.32 (0.06–1.02) vs 0.32 (0.11–0.83), *p* = 0.927] levels. Similar results of the plasma IFN-γ (12.82 (4.63–29.96) vs 10.22 (1.91–18.62), *p* = 0.220) and GM-CSF [0.40 (0.09–0.87) vs 0.27 (0.09–0.63), *p* = 0.316] levels were found between the higher percentage of PD-L1^+^ NK cells group and the lower percentage of PD-L1^+^ NK cells group.

## Discussion

Currently, there is an apparent lack of an accurate biomarker for predicting the prognosis of sepsis [15]. The present results have shown that the percentage of PD-L1^+^ NK cells may serve as a prognostic factor or marker for patients with sepsis as defined by the sepsis-3 criteria. This was based on the following findings and arguments: (1) the percentage of PD-L1^+^ NK cells of sepsis patients who died within 28 days was significantly higher than that of those who survived, (2) multivariate logistic regression analysis showed that the percentage of PD-L1^+^ NK cells and SOFA scores were independent risk factors for 28-day mortality, (3) the 28-day mortality was higher and the survival time was shorter in patients with a high percentage of PD-L1^+^ NK cells at the time of inclusion, and (4) septic patients with bloodstream infection showed a significant increase in percentage of PD-L1^+^ NK cells in comparison with patients with non-bloodstream infection.

Several previous studies have reported that the number of NK cells in the peripheral blood of sepsis patients is significantly higher than that of healthy individuals [16–18]. On the other hand, Boomer et al. [19] and Holub et al. [20] had reported decreased number of NK cells in the blood of patients at 24 h after the onset of sepsis. This phenomenon was thought to be related to the increased incidence of secondary infection [21] and sepsis encephalopathy [22]. The multi-faceted effects of NK cells have led to contradictory outcomes in patients with sepsis. Separately, it was reported that the expression levels of positive activated molecule NK62D, TLR-2/4/9, and CD69 on NK cells and plasma IFN-γ in sepsis patients were significantly higher than those in healthy controls [23]. Feng et al. [10] found that the proportion of NK cells in peripheral blood and positively activated molecule NKP30 were associated with 28-day mortality. Failure to take into account the surface inhibitory receptors of NK cells in current studies might have limited a fuller understanding on the role of NK cells in human sepsis. The present results corroborate that of Feng et al. [10] and confirm the predictive effect of NK cell count on 28-day mortality of sepsis patients. However, the present results have extended that when co-signaling molecules such as PD-1, CD28, PD-L1, and CD86 in NK cells were added into the multivariate regression analysis, the percentage of PD-L1^+^ NK cells was considered as an independent risk factor for 28-day mortality, suggesting that the inhibitory receptor, such as PD-L1 in NK cells might predict the prognosis of patients with sepsis as compared to the NK cell counts. To the best of our knowledge, this is the first study investigating the prognostic role of percentage of PD-L1^+^ NK cells in human sepsis.

Additionally, we show in this study that the percentage of PD-L1^+^ NK cells is positively correlated with APACHE II and SOFA scores on days 2, 3, and 6, suggesting that percentage of PD-L1^+^ NK cells is a reliable factor or marker to predict the severity of patients’ disease and organ damage. As opposed to the study by Neo et al. [24], no correlation was detected between the NK cell count/percentage of PD-L1^+^ NK cells and IFN-γ or GM-CSF in the current study. This may be due to the lack of dynamic changes in the two cytokines levels before or after changes in the NK cell count/percentage of PD-L1^+^ NK cells, and more importantly, NK cells may not be the only cellular source of plasma IFN-γ and GM-CSF. Indeed, plasma IFN-γ and GM-CSF may also be derived from other immune cells such as T lymphocytes. Of course, the insufficient sample size of two cytokines is also one of the reasons for this difference. The current study also found that the percentage of PD-L1^+^ NK cells was significantly higher in bloodstream-infected patients than in those without bloodstream infections. Thus, we speculated that increased percentage of PD-L1^+^ NK cells might lead to immune dysfunction, and local infection might easily invade the blood circulation, leading to poor clinical prognosis. It remains to be further confirmed, such as by in vitro experiments, if the susceptibility of high percentage of PD-L1^+^ NK cells to bloodstream infection is related to the secretion of IFN-γ and GM-CSF.

Furthermore, CRP and PCT levels at the time of inclusion did not predict 28-day mortality, but their levels after 3 days were predictive of prognosis, which is similar to the study by Guo et al. [25]. These results indicated that the percentage of PD-L1^+^ NK cells could predict the prognosis of sepsis patients earlier than conventional inflammatory markers, such as CRP and PCT. The AUC analysis showed that the percentage of PD-L1^+^ NK cells was similar to commonly used clinical scoring APACHE II and SOFA scores, NK counts, MFI of CD28 in NK, CRP d3, PCT d3, and PCT d5 in predicting the 28-day mortality.

In addition, two independent risk factors of regression analysis were constructed into a prediction model-SOFA+NK PD-L1 model. Its performance in predicting 28-day mortality was significantly better than APACHE II and SOFA scores and any other single indicator. When the cutoff = 0.1241, the sensitivity was 90.00%, specificity was 58.54%, Youden index was 48.54%, PPV was 44.27%, and NPV was 94.12%, for predicting 28-day mortality respectively. The SOFA+NK PD-L1 model significantly improves the AUC of SOFA scores (0.727–0.808). The evaluation items of the SOFA score include the related indexes to evaluate the function of the nervous system, blood system, circulatory system, respiratory system, liver, and kidney, but exclude the immune function. The current results suggested that the inclusion of indicators reflecting the immune function into the SOFA scoring system might further optimize the predictive efficacy of SOFA scores.

Nevertheless, the present study has a few limitations. First, this is a single-center study, the total number of patients enrolled was small, and the conclusion is not verified by external datasets. Secondly, there was only one time point at which our samples were collected. As the immune status of the NK cells varied with the passage of time, it would be ideal for analyzing the samples collected at continuous time points. Thirdly, the percentage of PD-L1^+^ NK cells that affected the prognosis of patients with sepsis has yet to be ascertained.

## Conclusions

The percentage of PD-L1^+^ NK cells at admission serves as a novel prognostic biomarker for predicting mortality and contributes to improve the predictive capacity of SOFA score in patients with sepsis. These findings encouraged further efforts in exploring the individualized treatment strategy based on risk stratification according to the percentage of PD-L1^+^ NK cells and SOFA score.

## Data Availability

The datasets generated and analyzed during the current study are available from the corresponding author upon reasonable request.

## Abbreviations

APACHE II: Acute Physiology and Chronic Health Evaluation II
AUC: Area under the receiver operating characteristic curve
CI: Confidence interval
CRP: C-reactive protein
EDTA: Ethylenediaminetetraacetic acid
GCS: Glasgow Coma Scale
GM-CSF: Granulocyte-macrophage colony-stimulating factor
HR: Hazard ratio
ICU: Intensive care unit
IFN-γ: Interferon-γ
Lac: Lactate
MAP: Mean arterial pressure
MFI: Mean of fluorescence intensity
NK: Natural killer
NPV: Negative predictive value
OR: Odds ratio
PCT: Procalcitonin
PD-1: Programmed cell death 1 receptor
PD-L1: Programmed cell death-ligand 1
PPV: Positive predictive value
ROC: Receiver operating characteristic
SOFA: Sepsis-related organ failure assessment
WBC: White blood cell
